# Amyloid-clearing proteins and their epigenetic regulation as a therapeutic target in Alzheimer’s disease

**DOI:** 10.3389/fnagi.2014.00235

**Published:** 2014-09-17

**Authors:** Natalia N. Nalivaeva, Nikolai D. Belyaev, Caroline Kerridge, Anthony J. Turner

**Affiliations:** ^1^School of Molecular and Cellular Biology, Faculty of Biological Sciences, University of LeedsLeed, UK; ^2^I.M.Sechenov Institute of Evolutionary Physiology and BiochemistrySt. Petersburg, Russia; ^3^Neurodegeneration DHT, Lilly, Erl Wood ManorWindlesham, Surrey, UK

**Keywords:** amyloid peptide, APP, AICD, endothelin-converting enzyme, histone deacetylases, insulin-degrading enzyme, neprilysin, transthyretin

## Abstract

Abnormal elevation of amyloid β-peptide (Aβ) levels in the brain is the primary trigger for neuronal cell death specific to Alzheimer’s disease (AD). It is now evident that Aβ levels in the brain are manipulable due to a dynamic equilibrium between its production from the amyloid precursor protein (APP) and removal by amyloid clearance proteins. Clearance can be either enzymic or non-enzymic (binding/transport proteins). Intriguingly several of the main amyloid-degrading enzymes (ADEs) are members of the M13 peptidase family (neprilysin (NEP), NEP2 and the endothelin converting enzymes (ECE-1 and -2)). A distinct metallopeptidase, insulin-degrading enzyme (IDE), also contributes to Aβ degradation in the brain. The ADE family currently embraces more than 20 members, both membrane-bound and soluble, and of differing cellular locations. NEP plays an important role in brain function terminating neuropeptide signals. Its decrease in specific brain areas with age or after hypoxia, ischaemia or stroke contribute significantly to the development of AD pathology. The recently discovered mechanism of epigenetic regulation of NEP (and other genes) by the APP intracellular domain (AICD) and its dependence on the cell type and APP isoform expression suggest possibilities for selective manipulation of NEP gene expression in neuronal cells. We have also observed that another amyloid-clearing protein, namely transthyretin (TTR), is also regulated in the neuronal cell by a mechanism similar to NEP. Dependence of amyloid clearance proteins on histone deacetylases and the ability of HDAC inhibitors to up-regulate their expression in the brain opens new avenues for developing preventive strategies in AD.

## Introduction

**Figure 1:**
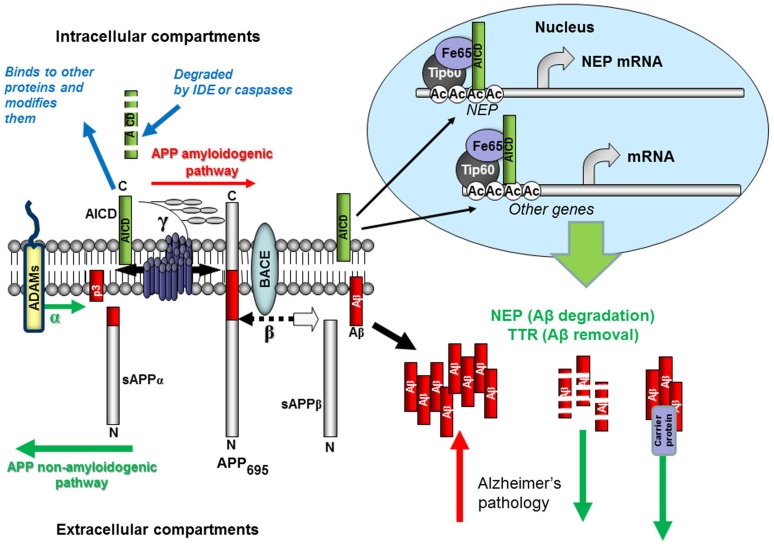
**Schematic representation of Aβ production and removal from the brain**. The proteolytic processing of the large, transmembrane, APP occurs in two distinct amyloidogenic and non-amyloidogenic pathways. The amyloidogenic pathway involves the sequential cleavage of APP by an aspartic proteinase, β-secretase, which releases a soluble ectodomain (sAPPβ) and the C-terminal fragment CTF99. This, in turn, is cleaved by another aspartic proteinase, γ-secretase, generating the transcriptional regulator APP intracellular domain (AICD), and releasing the 39-42 amino acid amyloid-β peptide (Aβ). Due to its very high ability for aggregation, Aβ forms dimers, trimers and higher level oligomers which are toxic to cells and cause neuronal death. Formation of amyloid plaques from Aβ aggregates in complex with other proteins is a hallmark of AD but is considered as a scavenging process. In the non-amyloidogenic pathway APP molecules are cleaved at the α-secretase site within the Aβ-domain releasing a soluble ectodomain sAPPα and the C-terminal fragment CTF83. Proteolytic cleavage of CTF83 by γ-secretase releases AICD and p3 fragment whose functions are still unknown. The AICD fragment produced in the amyloidogenic pathway binds to a stabilizing factor Fe65 and in a complex with other factors (the histone acetyl transferase, Tip60, and a Mediator complex subunit Med12) can act as a transcription enhancer regulating expression of a variety of genes, including the Aβ-degrading enzyme neprilysin and clearance protein transthyretin (TTR). This process was found to be specific to the neuronal APP_695_ isoform. AICD produced in the non-amyloidogenic pathway and from other APP isoforms (APP_751_ and APP_770_) is most likely degraded (by some intracellular proteases, e.g., insulin-degrading enzyme and caspases). Soluble APP ectodomains, sAPPα and sAPPβ, have been shown to have neuroprotective properties.

Overproduction and accumulation in the brain of abnormally high concentrations of the amyloid-β (Aβ) peptide and its oligomers causing synaptic loss and neuronal cell death are considered among the principal pathological events underlying neurodegeneration and Alzheimer’s disease (AD; Hardy and Higgins, 1992; Walsh et al., 2002; Hardy, 2009). Aβ is produced by the sequential proteolytic cleavage of the membrane-bound amyloid precursor protein (APP) by an aspartic protease, β-secretase (BACE1), followed by the presenilin-dependent γ-secretase (for details see Figure 1). Under normal physiological conditions it has important neuronal functions including transcriptional regulation (Koudinov and Berezov, 2004; Barucker et al., 2014). However, in some individuals mutations in the APP gene, or in the presenilin genes (PS1 and PS2), cause an increase in relative abundance of Aβ42 and development of AD pathology at a relatively early age. Our understanding of the molecular mechanisms of AD pathology to date derive from studies of the families manifesting early-onset AD and from studying transgenic animals expressing human genes bearing these pathological mutations (Guerreiro et al., 2012; LaFerla and Green, 2012). However, all attempts to create an effective drug against AD pathology based exclusively on the amyloid cascade hypothesis have not been fruitful. This can be explained by the fact that the majority of AD cases are of the late-onset, sporadic form not linked to mutations of the major AD-related genes but caused by some other pathological changes in normal brain physiology which predispose to overproduction and accumulation of Aβ in the brain over the years. Among them are neuroinflammation, poor diet, toxic environment, brain trauma, hypoxia, stroke and mutations in a number of other neuronal genes which not only shift the whole neuronal metabolism towards accumulation of Aβ (Karch et al., 2012) but also affect its intrinsic clearance both via transport and perfusion mechanisms or proteolytic cleavage.

It is now becoming more evident that Aβ levels in the brain are manipulable due to a dynamic equilibrium between its production from APP and removal by a cohort of amyloid clearance proteins which can be either of enzymic nature (proteases) or non-enzymic (binding/transport proteins) (Figure 1). The number of enzymes capable of proteolytic degradation of Aβ discovered to date is rapidly growing and currently the family of amyloid-degrading enzymes (ADEs) embrace more than 20 members both membrane-bound and soluble, and of differing cellular and subcellular locations (for review see Marr and Spencer, 2010; Nalivaeva et al., 2012a,c; Grimm et al., 2013; Pacheco-Quinto et al., 2013). Intriguingly several of the main ADEs are members of the M13 peptidase family (neprilysin (NEP), NEP2 and endothelin converting enzymes (ECE-1 and ECE-2)). Another metallopeptidase from the M16 family, insulin-degrading enzyme (IDE), also plays a significant role in Aβ degradation in the brain (Leissring et al., 2003; Qiu and Folstein, 2006). All these enzymes have been mostly studied in recent years with regard to their role in AD pathology or as therapeutic targets. This review will focus on the mechanisms regulating expression of their genes *in vivo* and *vitro*.

The amyloid binding/transport proteins which interact with Aβ and modulate its solubility, transport, clearance, degradation, and fibril formation are now widely studied. The serum amyloid P component (SAP), as well as apolipoprotein J (clusterin), have been described as the main Aβ-binding proteins while various apolipoproteins (apoA-IV, apoE, and apoA-I), as well as albumin (HSA) and fibulin were identified as minor contributors (Calero et al., 2012; Yu and Tan, 2012). Despite being a minor contributor to Aβ-binding in the plasma, apoE (especially the apoE4) is considered as one of the major contributors to Aβ clearance in the brain and is the major genetic risk factor for development of late-onset AD (for review see Hauser and Ryan, 2013). It has been suggested the ApoE/LRP1-mediated clearance of Aβ across the blood brain barrier (BBB) is the key event in the regulation of Aβ transport from brain to periphery which suggests that direct targeting of this process at the BBB could have potential in the treatment of late-onset AD (Martiskainen et al., 2013).

Another group of transport proteins, namely the ATP-binding cassette (ABC) transporters, which are localized on the surface of brain endothelial cells of the BBB and brain parenchyma, also contribute to Aβ clearance. Among these transporters the most important role in Aβ binding and transport is ascribed to ABCB1 (P-glycoprotein, P-gp), ABCG2 (breast cancer resistant protein, BCRP), ABCC1 (multidrug resistance protein 1, MRP1), and the cholesterol transporter ABCA (Abuznait and Kaddoumi, 2012). Recently special attention has been paid to transthyretin (TTR, formely known as thyroxine binding prealbumin) which can suppress the AD phenotype in transgenic animal models and reduce cerebral Aβ deposition (Li et al., 2013). Although being amyloidogenic itself in peripheral tissues, in the brain it was shown to have an anti-Aβ-amyloidogenic effect and is considered to have potential therapeutic value (Buxbaum and Reixach, 2009). In the brain this protein is mainly expressed by the cells of choroid plexus (Sousa et al., 2007) but can also be detected and regulated in neuronal cells (Kerridge et al., 2014) and apart from the transport function it can contribute to regulation of important neuronal proteins e.g., 14-3-3ζ protein and neuropeptide Y (Nunes et al., 2006; Vieira and Saraiva, 2013) and brain functions, including memory (Brouillette and Quirion, 2008; Vieira and Saraiva, 2014). TTR has been one of the proteins of interest in our recent research and the mechanisms of its epigenetic regulation were shown to be similar to those of an amyloid-degrading peptidase, NEP (Kerridge et al., 2014). Recent data also suggest that TTR expression in neuronal cells is regulated by heat shock factor 1 (HSF1) which is the major regulator of cellular stress responses (Wang et al., 2014a).

Within neuronal cells abnormal accumulation of Aβ might also result from the impairment of the two major protein quality control systems providing clearance of misfolded, aggregated or long-lived proteins and represented by autophagy-lysosome and ubiquitin-proteasome systems (for review see Lee et al., 2013). One of the lysosomal peptidases, namely cathepsin B, was shown to degrade Aβ and its insufficient expression or inactivation caused by its endogenous inhibitor cystatin C (CysC) could lead to Aβ accumulation (Wang et al., 2012). Deficit of another lysosomal enzyme, sialidase NEU1, also leads to accumulation and amyloidogenic processing of an oversialylated APP in lysosomes, and extracellular release of Aβ peptides by excessive lysosomal exocytosis (Annunziata et al., 2014). Reduced proteasome activity was demonstrated to increase levels of intracellular and secreted Aβ while accumulation of improperly degraded Aβ products in the lysosomes causes lysosomal disruption and cell death (Agholme et al., 2012). In turn, accumulation of Aβ oligomers in cells causes proteasome inhibition leading to cell death (Tseng et al., 2008). The oligomeric Aβ can be transmitted from one neuron to another causing neurotoxicity and as such the drugs which stop the spread of intracellular toxic Aβ aggregates may also be useful for early prevention of developing AD pathology (Agholme and Hallbeck, 2014). Both proteasomal and lysosomal degradation systems can be impaired during life by a variety of pathological factors resulting in accumulation of Aβ and predisposing to late-onset AD (Cuanalo-Contreras et al., 2013).

Understanding the clearance mechanisms in the brain and their regulation will help us to design therapies allowing the brain to fight against undesirable accumulation of toxic protein aggregates and to prolong its normal functioning. Deciphering the mechanisms of regulation of the major ADEs, in particular of NEP, lead us to a conclusion that there are some common targets which can be treated simultaneously by the same compounds modulating expression of several proteins involved in amyloid clearance (for example NEP and TTR), and this can result in design of a therapeutic strategy towards improved amyloid clearance.

### Neprilysin (NEP) and related peptidases (NEP2, ECE-1, ECE-2)

One of the first ADEs in the brain to be identified was a cell-surface, zinc metallopeptidase now known as NEP although this enzyme was originally identified in the brush border membrane of the kidney, where it is very abundant playing a role in degrading circulating biologically active peptides (Kerr and Kenny, 1974). It has subsequently been rediscovered in several guises in the body, for example in B lymphocytes where it is known as the common acute lymphoblastic leukaemia antigen (CALLA, CD10) (Letarte et al., 1988), and in the brain where it was initially identified as the peptidase turning off neuropeptide (enkephalins, substance P) signaling (Roques et al., 1980; Matsas et al., 1983). NEP is also the primary enzyme hydrolysing members of the atrial natriuretic peptide family in the periphery.

NEP preferentially cleaves small peptides (up to about 50 amino acids) on the *N*-terminal side of hydrophobic residues and hence the Aβ peptide is efficiently hydrolysed as was first shown *in vitro* by Howell et al. (1995). Subsequently, the enzyme was shown to be a major activity degrading both Aβ40 and Aβ42 in the brain by examining Aβ degradation in NEP-deficient mice and through the use of potent NEP inhibitors such as thiorphan (Iwata et al., 2001; Shirotani et al., 2001). Viral gene delivery of NEP to the brain of AD transgenic mice also was shown to reduce amyloid pathology (Marr et al., 2003) and subsequent studies, including the application of a recombinant, brain-targeted NEP (Spencer et al., 2014), have confirmed and extended these observations on the critical nature of NEP to amyloid clearance (Leissring et al., 2003). However, some caution is needed in over-expressing human NEP as a therapeutic strategy since, in a Drosophila model, while reducing transgenic Aβ42 levels this was accompanied by axonal loss and a reduction in lifespan (Iijima-Ando et al., 2008). Over-expression of a Drosophila homologue of NEP also resulted in significant behavioral deficits (Bland et al., 2009). These changes probably are a consequence of the substrate promiscuity of NEP resulting in altered levels of neuropeptides. A strategy to overcome this problem has involved identifying critical catalytic residues and hence engineering the specificity of human NEP to selectively enhance its Aβ-degrading capacity (Pope et al., 2014; Webster et al., 2014). Functional profiling of several ADEs in elderly and AD patients’ brains has provided additional support for NEP as the major protease involved in Aβ degradation (Wang et al., 2010).

The human genome contains seven NEP-like genes, several of which have now also been implicated in Aβ degradation. The closest homologue to NEP, NEP2 (also known as membrane metalloendopeptidase-like protein (MMELP)), can also degrade Aβ40 and Aβ42 *in vitro* and *in vivo* and is recognized as an important ADE and hence possible therapeutic target (Hafez et al., 2011). However, there are significant substrate and inhibitor specificity differences between rodent and human NEP2 so some caution is needed in extrapolating data between species (Whyteside and Turner, 2008). Unlike NEP, NEP2 exists in alternatively spliced forms with differing subcellular distributions but little is known of factors affecting NEP2 location or expression levels. Both NEP and NEP2 expression are altered in mild cognitive impairment (MCI) subjects relative to non-impaired subjects in AD-susceptible regions (Huang et al., 2012). NEP2 enzymic activity was decreased in both MCI and AD and was positively associated with cognitive functions which suggested that NEP2 may serve as a preclinical biomarker for AD (Huang et al., 2012).

Two other NEP-like gene products that act as ADEs are the endothelin converting enzymes (ECE-1 and ECE-2). ECE-1 is mainly located in vascular endothelial cells where its principal role is to act as a biosynthetic enzyme in the formation of the vasoconstrictor peptide endothelin-1 from its precursor proendothelin-1. ECE-1 may also metabolize some other circulating peptides, e.g., bradykinin (Hoang and Turner, 1997). However, significant levels of ECE-1 are present in the CNS (Barnes and Turner, 1997) being involved in brain endothelin synthesis. It also plays a role in endosomal neuropeptide metabolism regulating peptide receptor recycling (Cottrell et al., 2009). Like NEP, ECE-1 can degrade Aβ *in vitro*, and *in vivo* transgenic studies have implicated the enzyme in physiological Aβ metabolism (Eckman and Eckman, 2005). The much less studied homologue ECE-2, originally identified by Emoto and Yanagisawa (1995) is also an excellent candidate ADE being mainly brain located. ECE-2 levels are reported to be increased in AD and it is up-regulated by Aβ itself (Palmer et al., 2009). ECE-2, which has an acidic pH optimum, is localized in secretory compartments and appears to be involved in protein processing events (Mzhavia et al., 2003), including some opioid peptides and thereby regulating opioid receptor activity (Gupta et al., 2014). Increased concentrations of both Aβ1-40 and Aβ1-42 are found in the brains of ECE-2 knockout mice (Eckman et al., 2006). Recently using SH-SY5Y cells overexpressing wild-type APP and pharmacological inhibition of endogenous ECE activity it was shown that ECE-1 and ECE-2 participate in the degradation of distinct pools of Aβ, one destined for secretion and the other being produced and degraded within the endosomal-autophagic-lysosomal pathways (Pacheco-Quinto and Eckman, 2013). Although ECE-1 regulates both pools of Aβ, ECE-2 regulates mainly the intracellular pool of the peptide, in particular in the autophagic vesicles, suggesting that therapeutic targeting of these enzymes might have different outcomes since it will affect different pools of Aβ (Leissring and Turner, 2013; Pacheco-Quinto and Eckman, 2013).

Consistent with the observations that AD patients have reduced cerebral blood flow preceding onset of dementia, Palmer and colleagues have been able to link these changes with Aβ-induced cerebral vasoconstriction increasing ECE-1 levels and hence ET-1 in a free radical-mediated process (Palmer et al., 2009, 2012, 2013). Modulating ECE-1 levels therefore could have opposing neuroprotective and neurotoxic effects through effects on both Aβ and ET-1 levels.

### Insulin-degrading enzyme (insulysin, IDE)

Another principal ADE, and the first to be identified (Kurochkin and Goto, 1994), is the IDE (insulysin). This is a zinc peptidase but structurally and mechanistically distinct from the NEP family and also showing distinct cellular and subcellular localization. A very diverse range of studies, from *in vitro* and cell-based studies through to animal models now support an involvement of IDE in amyloid clearance mechanisms (for review see Fernández-Gamba et al., 2009; Nalivaeva et al., 2012a). Amyloid peptides associated with British and Danish familial dementia were found among the specific IDE substrates (Morelli et al., 2005). As its name suggests, IDE was originally described as an enzyme implicated in insulin metabolism and therefore of potential significance in diabetes mellitus providing one of a number of links between diabetes and AD (Yang and Song, 2013; Haque and Nazir, 2014). Recently it was shown that IDE inhibitors might represent a new therapeutic strategy to treat type-2 diabetes (Maianti et al., 2014).

IDE has important substrates other than insulin and Aβ. In particular, IDE is involved in elimination of oxidized proteins in peroxisomes (Morita et al., 2000). It is also the principal proteinase degrading cytosolic APP intracellular domain (AICD) (Edbauer et al., 2002). Another physiological substrate for IDE is somatostatin, a neuropeptide that declines in aging and AD, which is also able to bind to and enhance IDE activity towards Aβ through an allosteric action (Ciaccio et al., 2009) and to upregulate NEP expression (Saito et al., 2005). Although it is primarily located in the cytosol, IDE is found in lesser amounts in mitochondria where Aβ can also be found (Leissring et al., 2004). Surprisingly, given that it lacks any known secretory signal, a small but significant proportion of IDE is secreted from cells through an unconventional protein secretion pathway (Zhao et al., 2009). This secreted IDE is functional in extracellular Aβ degradation and it appears to be routed via detergent-resistant membrane complexes into exosomes for secretion, along with Aβ (Bulloj et al., 2010).

The recent development of novel IDE activators and inhibitors (Cabrol et al., 2009; Leissring et al., 2010; Abdul-Hay et al., 2013; Maianti et al., 2014) combined with detailed structural studies of the enzyme (Im et al., 2007) should aid in unraveling the importance of IDE in amyloid metabolism and pathology, and as a therapeutic target.

### Other ADEs

A wide and diverse variety of other proteases of all main catalytic classes have been suggested as potential ADEs and their involvement in these processes has been reviewed elsewhere (e.g., Nalivaeva et al., 2012a) and will not be covered in detail here. These include some metallopeptidases (matrix metalloproteinases, glutamate carboxypeptidase II), serine proteases (plasmin, acylpeptide hydrolase), cysteine proteases (cathepsin B) and the aspartic protease, BACE-2. The latter enzyme was a particularly efficient ADE and able to degrade intracellular Aβ (Abdul-Hay et al., 2012). Although angiotensin-converting enzyme (ACE) was shown to be capable of Aβ cleavage (Hemming and Selkoe, 2005) mostly acting as a carboxydipeptidase and converting the more hydrophobic and toxic Aβ_42_ to Aβ_40_ (Zou et al., 2007), its role in AD pathology is still not clear. A recent study employing ACE overexpression in myelomonocytes of APP(SWE)/PS1(ΔE9) mice has convincingly demonstrated the protective effect of ACE against cognitive decline but this effect was most likely linked to an enhanced immune response, coupled with increased myelomonocytic expression of catalytically active ACE (Bernstein et al., 2014). The ACE homologue, ACE2, is a metallo-carboxypeptidase that converts Aβ_43_ to Aβ_42_ which can then be converted to Aβ_40_ by ACE (Liu et al., 2014). Hence, maintaining brain ACE2 and ACE activities might be important for preventing accumulation of more toxic Aβ species.

## Deficit of ADEs under pathological conditions and ageing

Ageing is considered as one of the major risk factors of late-onset AD. Although literature data demonstrate a link between AD pathology and the level of activity and expression of ADEs in the brain there are some contradictions in the reported results which might be caused by the source of the brain material used, its maintenance before the analysis and also by the techniques applied. Reduced NEP mRNA levels were reported in the hippocampus and temporal gyrus of AD patients suggesting a relationship between NEP activity and deficient degradation of Aβ peptide leading to high number of senile plaques in these brain areas (Yasojima et al., 2001). Similarly, reduced IDE levels were reported in the hippocampus of AD patients bearing the APOEɛ4 allele (Cook et al., 2003). It was also shown that NEP and IDE decline with age in human cortex and hippocampus and IDE is more oxidized in the hippocampus of AD patients compared to the cerebellum (Caccamo et al., 2005). Reduced activity of NEP was also observed in the cerebrospinal fluid (CSF) of demented patients with Lewy body pathology (Maetzler et al., 2010). On the contrary there were no differences reported in NEP mRNA levels in the temporal lobe of AD patients compared to age-matched controls although the authors observed some changes in the somatostatin system suggesting that this might impair NEP regulation and cause a deficit of its activity leading to Aβ accumulation (Gahete et al., 2010). Moreover, postmortem analysis of NEP activity by immunocapture-based fluorogenic assays revealed an increase in NEP and IDE activity in the frontal cortex from subjects of different ages and with different pathological stages of AD progression (Miners et al., 2009). Based on these observations the authors suggested that reduction in NEP and IDE activity is not the primary cause of Aβ accumulation in AD, but rather a late-stage phenomenon secondary to neurodegeneration. Our own data on rat brain demonstrate that NEP mRNA and protein levels in the cortex and hippocampus significantly decline with age being the highest in the first month of animal life when development of the nervous system and its neuronal networks is the most active. On the contrary, in the striatum, NEP expression remained at a relatively high level during advanced age (Nalivaeva et al., 2004, 2012c). Regarding the human brain there are no systematic data showing the dynamics of NEP or other ADEs expression in various brain structures with aging and in the cases of development of MCI and AD which would significantly contribute to our understanding of the role of these enzymes in normal human brain development, functioning and pathogenesis. The recently developed McGill-Thyl-APP transgenic mouse model of AD demonstrates significant down-regulation of IDE at the preclinical early stage of AD pathology which could make it a valuable model for exploring mechanisms to restore enzyme activity (Ferretti et al., 2011).

Among the factors which predispose to the development of late-onset AD are hypoxia, ischaemia, stroke, stress, diabetes, trauma and neuroinflammation. All of them were shown to affect amyloid metabolism and to some extent the levels of expression and activity of ADEs. The analysis of ischaemically damaged brains of patients demonstrated that ischaemic episodes trigger dysregulation of expression of a number of AD-related genes (Pluta et al., 2013). Monitoring plaque formation in real time in a mouse model has revealed that stroke can trigger accelerated amyloid deposition, most likely through interference with amyloid clearance pathways along interstitial fluid drainage routes (Garcia-Alloza et al., 2011). Our studies have demonstrated that experimental four-vessel occlusion ischaemia in rats resulted in down-regulation of NEP and ECE-1 as well as up-regulation of APP and BACE both in the cortex and hippocampus (Nalivaeva et al., 2004, 2005). On the contrary, levels of IDE were up-regulated after ischaemia both at the mRNA and protein levels (unpublished data). These findings strongly correlate with the changes in NEP and IDE expression patterns observed in rat thalamus after focal cerebral ischaemia (Hiltunen et al., 2009). Up-regulation of IDE mRNA was also observed in a permanent two-vessel occlusion ischaemia model in rats which was attenuated by administration of a natural product (Choto-san) widely used in Japan for treatment of vascular dementia (Hayashi et al., 2005). The apparent anti-dementia effects of this medicine have been shown to be due to its anti-hypertensive, free radical scavenging, and anti-excitotoxic effects (Watanabe et al., 2003). However, these authors used whole brain mRNA extracts which diminishes the value of their results in relation to the brain areas affected by AD. A different pattern of IDE regulation under ischaemic conditions was also observed in wild-type mice and mice with a deficient Toll-receptor system suggesting that the Toll/interleukin-1 receptor domain-containing adaptor inducing interferon-β (TRIF) pathway regulates IDE expression (Famakin et al., 2014).

Cerebrovascular pathology and ischaemia are the major factors leading to insufficient oxygen supply to the brain causing metabolic changes in neuronal cells predisposing to neurodegeneration and AD (Bazan et al., 2002). Previously we have demonstrated that hypoxia in neuroblastoma NB7 cells and rat primary cortical neurones lead to down-regulation of mRNA and protein levels of NEP as well as its enzyme activity (Fisk et al., 2007). We have also observed around 30% decrease in expression of ECE-1 under hypoxic conditions at the protein level, although at the level of ECE-1 mRNA there were no statistically significant changes (Fisk et al., 2006). These data correlate with reduced NEP activity in the cortex and hippocampus of rats subjected to prenatal hypoxia (Nalivaeva et al., 2012b; Wang et al., 2014b).

In the AD brain decreased levels of NEP expression were also observed along the vasculature suggesting its role in the development of cerebral amyloid angiopathy (CAA) in AD patients (Carpentier et al., 2002; Miners et al., 2006). Assessing the protective role of NEP in the vasculature it was shown that NEP can rescue cerebrovascular smooth muscle cells against Aβ-induced cell death and prevent complications of CAA (Miners et al., 2011). Along with the age-related decrease of NEP expression observed in neuronal cells, Apelt and colleagues have reported up-regulation of this enzyme in reactive astrocytes surrounding amyloid plaques in AD transgenic mice (Apelt et al., 2003) which is considered to be a compensatory reaction of a specific set of brain cells possessing a protective function. These observations stimulated a cascade of studies on possible mechanisms of NEP up-regulation as a strategy for AD treatment through stimulating clearance of Aβ peptides and/or their various aggregated forms.

## Pharmacological and epigenetic regulation of ADE genes

Various strategies have been suggested to increase expression and activity of ADEs, in particular of NEP, in the brain areas vulnerable to accumulation of amyloid burden and neuronal cell death. Developing targeted gene delivery techniques several viral vectors have been utilized including Sindbis viral (Hama et al., 2001), lentiviral (Marr et al., 2003) or adenoviral (Iwata et al., 2013) delivery which proved to be effective in reducing amyloid burden in treated brain areas. The last approach, which allowed global neuronal gene expression throughout the brains, utilized peripheral intravascular administration of the gene-targeted vector. Refining the methods of NEP delivery from the periphery to the brain, intra-peritoneal injections of a lentiviral vector expressing NEP fused with the ApoB transport domain was found to be effective in reducing levels of Aβ and the number of plaques in the brain of AD transgenic mice (Spencer et al., 2011). The optimal timing of NEP over-expression has also been examined suggesting that earlier up-regulation of NEP was more beneficial in alleviating AD symptoms in a mouse model (El-Amouri et al., 2008). In line with the attempts at transgenic over-expression of NEP in neuronal cells it was also shown that increased IDE expression can lead to reduced brain Aβ and delay of AD pathology (Leissring et al., 2003).

Delivery of NEP to peripheral tissues has also proved effective in reducing the deposits of islet amyloid polypeptide (IAPP) which contribute to type 2 diabetes. Thus, adenoviral delivery of NEP to the pancreas reduced amyloid deposits and cell death in the islets (Zraika et al., 2010). However, in this case, NEP did not act via degradation of IAPP but appeared to act by inhibiting the amyloid fibril formation through protein-protein interactions involving the enzyme active site, rather than by hydrolysing the peptide.

Because brain and plasma Aβ are considered to be in an equilibrium through transport mechanisms (Deane et al., 2004), Hersh and colleagues have investigated whether enhancing peripheral degradation of Aβ by NEP could reduce brain levels and associated pathology. Their reports suggest that in AD transgenic mice over-expression of NEP in erythrocytes or leukocytes leads to a reduced Aβ burden in the brain (Liu et al., 2007; Guan et al., 2009). Similar results were observed when NEP was over-expressed in skeletal muscle (Liu et al., 2009). An alternative strategy of expressing a secreted, soluble form of NEP in plasma through an adenovirus construct was also effective in clearing brain Aβ yet did not affect plasma levels of other peptide substrates of NEP such as bradykinin or substance P (Liu et al., 2010). Expressing NEP in plasma might provide a simple system allowing the monitoring of its long-term activity, and this approach has recently been re-examined by using albumin-fused NEP with extended half-life (Henderson et al., 2014). This study demonstrated that although NEP highly effectively degraded Aβ in the periphery it did not alter brain or CSF Aβ levels, suggesting that the peripheral sink hypothesis might not be valid and supporting earlier observations of Walker et al. (2013). However, the experiments in both studies have been performed in transgenic mice and non-human primates and the validity of these conclusions for human pathology still needs to be assessed.

Apart from gene-targeted manipulations of the expression of ADEs, a number of studies have aimed at pharmacological up-regulation of these enzymes and one of the earlier works reported that retinoic acid which induces cell differentiation was able to increase IDE expression and activity (Melino et al., 1996). Aiming at NEP regulation, Saido et al. have put forward a hypothesis that elevated levels of neuropeptide substrates of NEP could themselves up-regulate NEP via a feedback control mechanism. They hence screened a wide range of NEP substrates demonstrating that, of those tested, only somatostatin was capable of up-regulating NEP expression in primary neuronal cells in a mechanism possibly mediated through somatostatin receptor sub-types 2 or 4 (Saito et al., 2005). Utilization of somatostatin agonists as a possible therapeutic strategy for prevention of AD was recently reinforced by Saido (2013). An alternative strategy for pharmacological up-regulation of NEP was reported in a study analyzing the effects of a selective peroxisome proliferator activated receptor-δ (PPARδ) agonist, GW742, which activated the NEP promoter driving luciferase expression in transfected HEK293 cells (Kalinin et al., 2009). This treatment reduced plaque burden in 5xFAD mice which harbor 3 mutations in APP and 2 mutations in PS1 not only by NEP activation but also by increased expression of IDE. IDE expression was also shown to be up-regulated by norepinephrine via the β_2_ adrenergic receptor resulting in activation of Aβ uptake and degradation by microglia (Kong et al., 2010). Another brain metabolite, namely kynurenic acid, was reported to regulate NEP mRNA and protein levels in SH-SY5Y cells and mouse cortical neurones and to have a protective effect on cell survival which was thiorphan-dependent (Klein et al., 2013).

Although reports on the effect of the protein kinase C inhibitor and anticancer drug Gleevec on NEP up-regulation were to some extent contradictory (Eisele et al., 2007; Vázquez et al., 2009), our recent data confirmed that Gleevec can increase NEP expression in cell models (Kerridge et al., 2014). On the other hand, selective activation of protein kinase Cɛ (e.g., with bryostatin) was also shown to reduce Aβ levels through enhancing NEP expression and cell-surface activity (Lim and Alkon, 2014).

Analyzing the effects of such common drugs as statins on the status of ADEs in the brain Tamboli et al. (2010) demonstrated a potential positive effect of lovastatin and itraconazole on Aβ degradation and clearance via increased exosome-associated secretion of IDE by microglia. Lovastatin injections also stimulated release of IDE in the circulation of mice. However, in another study using a transgenic mouse model the authors failed to observe any effects of simvastatin on NEP activity (Papadopoulos et al., 2014). Among lipid species, docosahexaenoic acid (DHA) was also shown to up-regulate IDE while palmitic acid had an opposite effect (Du et al., 2010).

A variety of other strategies to enhance activity levels of NEP have also been examined including epigenetic approaches (see below), as well as natural products that can upregulate the enzyme (green tea catechins, curcumin, flavones, etc.) (Melzig and Janka, 2003; Ayoub and Melzig, 2006; Kiss et al., 2006; Deng et al., 2014; Fujiwara et al., 2014). Green tea catechins were shown to be preventive in AD animal models via up-regulation of NEP (Lim et al., 2013). Human adipose tissue-derived mesenchymal stem cells have been shown to secrete exosomes carrying functionally active NEP (Katsuda et al., 2013) and neuronally differentiated human umbilical cord mesenchymal stem cells transplanted into APP transgenic mice reduced Aβ levels and enhanced both NEP and IDE levels, as well as cognitive function, raising the prospect of future cell therapy for AD (Yang et al., 2013). Recently a non-invasive physiologically relevant way to up-regulate NEP expression was examined in an animal model demonstrating that enriched environment has a protective effect against Aβ accumulation and age-related cognitive decline (Mainardi et al., 2014) supporting earlier observations that brain NEP is upregulated under such conditions (Lazarov et al., 2005).

## Role of APP in regulation of ADE genes

Apart from Aβ and N-terminal products released from APP during its proteolytic processing via γ-secretase cleavage (see Figure 1) there are several C-terminal fragments or AICDs. Their formation is similar to the cleavage of the Notch protein, which liberates an intracellular domain (NICD) capable of translocating to the nucleus and regulating gene transcription (Schroeter et al., 1998). AICD was also proposed to act as a transcriptional regulator (Passer et al., 2000) and by using luciferase reporter-based assays, it was shown that AICD can form a transcriptionally active complex with the nuclear adaptor (and AICD stabilizing protein) Fe65 and the histone acetyltransferase Tip60 (Cao and Südhof, 2001).

Since that first report of the transcriptional activity of the C-terminal fragment released from APP cleavage by γ-secretase, the transcriptional activity of AICD has been intensively studied (for review, see Beckett et al., 2012; Konietzko, 2012; Pardossi-Piquard and Checler, 2012) and the number of genes suggested to be regulated by AICD is steadily increasing. However, to date, only the NEP gene has unequivocally been shown to be functionally regulated by AICD (Pardossi-Piquard et al., 2005; Belyaev et al., 2009). Moreover, it has been established that only the AICD derived from the neuronal APP_695_ but not from APP_751_ or APP_770_ in a β-secretase-dependent pathway is accumulated in the nucleus and up-regulates NEP expression (Belyaev et al., 2010). Mechanistically at the transcriptional level up-regulation of NEP expression by AICD involves the binding of AICD to the MED12 unit of the Mediator RNA polymerase II complex (Xu et al., 2011). This finding provides a missing functional link between nuclear AICD binding and its transcriptional activity (Turner et al., 2011) and allowed the validation of additional AICD-dependent genes such as aquaporin-1, MICAL2 and Fibronectin-1 (Xu et al., 2011).

Using two neuroblastoma cell lines, SH-SY5Y characterized by very low levels of NEP expression, and NB7 which produce a significant amount of NEP we have demonstrated that in the case of low NEP-expressing cells the *NEP* promoter is occupied by the histone-deacetylase HDAC1 while in NB7 cells by AICD. Treating SH-SY5Y cells with HDAC inhibitors trichostatin A or valproic acid (VA) we were able to up-regulate levels of NEP expression (Belyaev et al., 2009). VA is chiefly known as a highly successful and widely used antiepileptic drug but its efficacy in neurodegenerative disease has been much less studied (Nalivaeva et al., 2009), although there are several recent reports of its beneficial effects in reducing amyloid accumulation and improving cognitive function in AD mice (Qing et al., 2008), and amyloid clearance via microglial phagocytosis (Smith et al., 2010). VA also up-regulates NEP in the cortex and hippocampus and restores memory deficit caused by hypoxia in rats (Nalivaeva et al., 2012b) and in transgenic mice subjected to prenatal hypoxia (Wang et al., 2014b). The potential of VA and other HDAC inhibitors in the treatment of neurodegeneration has been further strengthened by the recent report that inhibitors of class 1 HDACs reverse contextual memory deficits in an AD mouse model (Kilgore et al., 2010), a process termed “epigenetic memory rescue”. Others have also supported the potential value of VA in AD as a mechanism to “untie tangles” coupled with its ability to induce neurogenesis of neural progenitor/stem cells both *in vitro* and *in vivo* via multiple signaling pathways including up-regulation of the nerve growth factor gene (Nalivaeva et al., 2009; Tariot and Aisen, 2009; Noh and Seo, 2014). There is clearly scope for more robust clinical trials on selective HDAC inhibitors in AD, particularly at earlier stages in disease progression. Given the prolonged and successful use of VA clinically in the treatment of epilepsy, there are grounds for more detailed retrospective epidemiological surveys in relation to AD incidence in valproate-users and control patients.

Since AICD is rapidly degraded within the cytoplasm, in particular by IDE (Cupers et al., 2001; Edbauer et al., 2002) treatment of cells with IDE inhibitors or an alkalising agent such as NH_4_Cl (Vingtdeux et al., 2007) was proved to be able to stabilize AICD and its nuclear pool resulting in increased NEP expression and activity and reduced Aβ levels in the cell medium (Kerridge et al., 2014). Apart from degradation, Aβ-sequestration by clearance proteins also precludes Aβ oligomerization and plaque formation. A major Aβ-binding protein in the brain is TTR (Du et al., 2012), which suppresses Aβ aggregation, facilitates its clearance and prevents Aβ-induced cytotoxicity (Tsuzuki et al., 2000; Brouillette et al., 2012; Li et al., 2013). Increased levels of TTR have been observed in transgenic mice over-expressing mutant APP (Stein and Johnson, 2002) but whether this effect was mediated by AICD and is APP isoform-dependent has not been investigated. Recently we have reported that TTR is regulated by AICD and HDAC similarly to NEP (Kerridge et al., 2014).

Among therapeutic strategies to improve Aβ clearance in the brain special attention has been paid to a recently reported effect of a retinoid X receptors (RXR) agonist bexarotene also used as an anti-cancer drug in patients with cutaneous T-cell lymphoma (Cramer et al., 2012). Oral administration of this drug to mice with AD pathology caused a rapid enhanced clearance of soluble Aβ which resulted within a few days in a reduced amyloid plaque area and in rapid reversal of cognitive, social, and olfactory deficits. Such fast clearance of Aβ in the brain had an ApoE-dependent character although there might be other mechanisms involved and more studies are need to evaluate the clinical value of this drug in AD (LaFerla, 2012; Strittmatter, 2012). Bexarotene also blocks calcium-permeable ion channels formed by Aβ peptides by competing with a cholesterol-binding domain in Aβ (Fantini et al., 2014).

## Future perspectives

As suggested above, epigenetic regulation of AD-related genes is now considered a plausible strategy for designing new therapies against late-onset AD. For example, simultaneous up-regulation of NEP and TTR gene expression by Gleevec might provide a platform for designing a strategy towards enhancement of amyloid clearance in the ageing brain via different mechanisms. Epigenetic regulation of the clusterin gene expression can also play an important role in AD pathogenesis via inhibition of Aβ aggregation (Li et al., 2014).

Although several compounds have been discarded on the grounds of limited clinical efficacy, all major clearance-related approaches still hold promise. Among some drug candidates advanced to Phase III trials are anti-Aβ antibodies, metal complexing agents, ginseng extracts, and intravenous immunoglobulins (Kurz and Perneczky, 2011). Recent data, including our own reports, strongly support the possibility that clearance-related strategies have the potential for protecting neuronal cells and cognitive functions via reducing the levels of soluble and particularly toxic Aβ species in the brain.

With all the advances in our knowledge of amyloid metabolism and the increase in number of ADEs that have been identified in multiple cell types and brain regions acting co-ordinately to regulate Aβ levels, it is still very surprising that relatively little is known about the precise physiological role of this peptide. Considering that in the cells there is a very well defined and tuned mechanism of Aβ production and removal one can expect that this peptide might have an important role which, when disturbed, can lead to the disease. New functions of Aβ continue to emerge, for example, even as a regulator of gene transcription (Maloney and Lahiri, 2011; Barucker et al., 2014). Recent strategies in drug design could be more successful if more effort had been put into the study of normal physiology of this peptide and its precursor protein APP (Nalivaeva and Turner, 2013).

Taking into account that AD pathology develops over many years, early manipulation of ADEs might be essential to prevent amyloid deposition and disease progression. However, the efficacy of any preventive treatment will be strongly driven by the development of early diagnosis and pre-clinical markers of AD. Recent systematic studies of blood protein biomarkers have shown potential in prediction and monitoring the progression of AD (Kiddle et al., 2014; Sattlecker et al., 2014). Levels of expression and activity of ADEs, e.g., in blood serum, might be of some importance but more systematic analysis is required to prove any correlation between these enzymes and disease progression. Since AD is a multifactorial disease a single focus for diagnostics or treatment is unlikely to be productive so a combination therapy targeting various links in the chain of pathological events leading to AD is likely to be optimal. Combining the efforts in gaining new knowledge about various protective mechanisms and the ways of their enhancement is the key to success.

## Conflict of interest statement

The authors declare that the research was conducted in the absence of any commercial or financial relationships that could be construed as a potential conflict of interest.

## References

[B1] Abdul-Hay S. O. Lane A. L. Caulfield T. R. Claussin C. Bertrand J. Masson A. (2013). Optimization of peptide hydroxamate inhibitors of insulin-degrading enzyme reveals marked substrate-selectivity. J. Med. Chem. 56, 2246–2255 10.1021/jm301280p 23437776 PMC10165945

[B2] Abdul-Hay S. O. Sahara T. McBride M. Kang D. Leissring M. A. (2012). Identification of BACE2 as an avid β-amyloid-degrading protease. Mol. Neurodegener. 7:46 10.1186/1750-1326-7-46 22986058 PMC3470943

[B3] Abuznait A. H. Kaddoumi A. (2012). Role of ABC transporters in the pathogenesis of Alzheimer’s disease. ACS Chem. Neurosci. 3, 820–831 10.1021/cn300077c 23181169 PMC3504479

[B4] Agholme L. Hallbeck M. (2014). Getting rid of intracellular Aβ- loss of cellular degradation leads to transfer between connected neurons. Curr. Pharm. Des. 20, 2458–2468 23859554 10.2174/13816128113199990501

[B5] Agholme L. Hallbeck M. Benedikz E. Marcusson J. Kågedal K. (2012). Amyloid-β secretion, generation and lysosomal sequestration in response to proteasome inhibition: involvement of autophagy. J. Alzheimers Dis. 31, 343–358 10.3233/JAD-2012-120001 22555375

[B6] Annunziata I. Patterson A. Helton D. Hu H. Moshiach S. Gomero E. (2014). Lysosomal NEU1 deficiency affects amyloid precursor protein levels and amyloid-β secretion via deregulated lysosomal exocytosis. Nat. Commun. 4:2734 10.1038/ncomms3734 24225533 PMC4015463

[B7] Apelt J. Ach K. Schliebs R. (2003). Aging-related down-regulation of neprilysin, a putative β-amyloid-degrading enzyme, in transgenic Tg2576 Alzheimer-like mouse brain is accompanied by an astroglial upregulation in the vicinity of β-amyloid plaques. Neurosci. Lett. 339, 183–186 10.1016/s0304-3940(03)00030-2 12633883

[B8] Ayoub S. Melzig M. F. (2006). Induction of neutral endopeptidase (NEP) activity of SK-N-SH cells by natural compounds from green tea. J. Pharm. Pharmacol. 58, 495–501 10.1211/jpp.58.4.0009 16597367

[B9] Barnes K. Turner A. J. (1997). The endothelin system and endothelin-converting enzyme in the brain: molecular and cellular studies. Neurochem. Res. 22, 1033–1040 10.1023/A:1022435111928 9239759

[B10] Barucker C. Harmeier A. Weiske J. Fauler B. Albring K. F. Prokop S. (2014). Nuclear translocation uncovers the amyloid Peptide Aβ42 as a regulator of gene transcription. J. Biol. Chem. 289, 20182–20191 10.1074/jbc.M114.564690 24878959 PMC4106333

[B11] Bazan N. G. Palacios-Pelaez R. Lukiw W. J. (2002). Hypoxia signaling to genes: significance in Alzheimer’s disease. Mol. Neurobiol. 26, 283–298 10.1385/mn:26:2-3:283 12428761

[B12] Beckett C. Nalivaeva N. N. Belyaev N. D. Turner A. J. (2012). Nuclear signalling by membrane protein intracellular domains: the AICD enigma. Cell. Signal. 24, 402–409 10.1016/j.cellsig.2011.10.007 22024280

[B13] Belyaev N. D. Kellett K. A. Beckett C. Makova N. Z. Revett T. J. Nalivaeva N. N. (2010). The transcriptionally active amyloid precursor protein (APP) intracellular domain is preferentially produced from the 695 isoform of APP in a β-secretase-dependent pathway. J. Biol. Chem. 285, 41443–41454 10.1074/jbc.M110.141390 20961856 PMC3009870

[B14] Belyaev N. D. Nalivaeva N. N. Makova N. Z. Turner A. J. (2009). Neprilysin gene expression requires binding of the amyloid precursor protein intracellular domain to its promoter: implications for Alzheimer disease. EMBO Rep. 10, 94–100 10.1038/embor.2008.222 19057576 PMC2613207

[B15] Bernstein K. E. Koronyo Y. Salumbides B. C. Sheyn J. Pelissier L. Lopes D. H. (2014). Angiotensin-converting enzyme overexpression in myelomonocytes prevents Alzheimer’s-like cognitive decline. J. Clin. Invest. 124, 1000–1012 10.1172/jci66541 24487585 PMC3934162

[B17] Bland N. D. Robinson P. Thomas J. E. Shirras A. D. Turner A. J. Isaac R. E. (2009). Locomotor and geotactic behavior of Drosophila melanogaster over-expressing neprilysin 2. Peptides 30, 571–574 10.1016/j.peptides.2008.10.020 19038301

[B18] Brouillette J. Caillierez R. Zommer N. Alves-Pires C. Benilova I. Blum D. (2012). Neurotoxicity and memory deficits induced by soluble low-molecular-weight amyloid-β1-42 oligomers are revealed in vivo by using a novel animal model. J. Neurosci. 32, 7852–7861 10.1523/JNEUROSCI.5901-11.2012 22674261 PMC6620963

[B19] Brouillette J. Quirion R. (2008). Transthyretin: a key gene involved in the maintenance of memory capacities during aging. Neurobiol. Aging 29, 1721–1732 10.1016/j.neurobiolaging.2007.04.007 17512093

[B20] Bulloj A. Leal M. C. Xu H. Castaño E. M. Morelli L. (2010). Insulin-degrading enzyme sorting in exosomes: a secretory pathway for a key brain amyloid-β degrading protease. J. Alzheimers Dis. 19, 79–95 10.3233/JAD-2010-1206 20061628 PMC4414343

[B21] Buxbaum J. N. Reixach N. (2009). Transthyretin: the servant of many masters. Cell. Mol. Life Sci. 66, 3095–3101 10.1007/s00018-009-0109-0 19644733 PMC4820353

[B22] Cabrol C. Huzarska M. A. Dinolfo C. Rodriguez M. C. Reinstatler L. Ni J. (2009). Small-molecule activators of insulin-degrading enzyme discovered through high-throughput compound screening. PLoS One 4:e5274 10.1371/journal.pone.0005274 19384407 PMC2668070

[B23] Caccamo A. Oddo S. Sugarman M. C. Akbari Y. LaFerla F. M. (2005). Age- and region-dependent alterations in Aβ-degrading enzymes: implications for Aβ-induced disorders. Neurobiol. Aging 26, 645–654 10.1016/j.neurobiolaging.2004.06.013 15708439

[B24] Calero M. Rostagno A. Ghiso J. (2012). Search for amyloid-binding proteins by affinity chromatography. Methods Mol. Biol. 849, 213–223 10.1007/978-1-61779-551-0_15 22528093 PMC3665336

[B25] Cao X. Südhof T. C. (2001). A transcriptively active complex of APP with Fe65 and histone acetyltransferase Tip60. Science 293, 115–120 10.1126/science.1058783 11441186

[B26] Carpentier M. Robitaille Y. DesGroseillers L. Boileau G. Marcinkiewicz M. (2002). Declining expression of neprilysin in Alzheimer disease vasculature: possible involvement in cerebral amyloid angiopathy. J. Neuropathol. Exp. Neurol. 61, 849–856 12387451 10.1093/jnen/61.10.849

[B27] Ciaccio C. Tundo G. R. Grasso G. Spoto G. Marasco D. Ruvo M. (2009). Somatostatin: a novel substrate and a modulator of insulin-degrading enzyme activity. J. Mol. Biol. 385, 1556–1567 10.1016/j.jmb.2008.11.025 19073193

[B28] Cook D. G. Leverenz J. B. McMillan P. J. Kulstad J. J. Ericksen S. Roth R. A. (2003). Reduced hippocampal insulin-degrading enzyme in late-onset Alzheimer’s disease is associated with the apolipoprotein E-ɛ4 allele. Am. J. Pathol. 162, 313–319 10.1016/s0002-9440(10)63822-9 12507914 PMC1851126

[B29] Cottrell G. S. Padilla B. E. Amadesi S. Poole D. P. Murphy J. E. Hardt M. (2009). Endosomal endothelin-converting enzyme-1: a regulator of β-arrestin-dependent ERK signaling. J. Biol. Chem. 284, 22411–22425 10.1074/jbc.M109.026674 19531493 PMC2755963

[B30] Cramer P. E. Cirrito J. R. Wesson D. W. Lee C. Y. Karlo J. C. Zinn A. E. (2012). ApoE-directed therapeutics rapidly clear β-amyloid and reverse deficits in AD mouse models. Science 335, 1503–1506 10.1126/science.1217697 22323736 PMC3651582

[B31] Cuanalo-Contreras K. Mukherjee A. Soto C. (2013). Role of protein misfolding and proteostasis deficiency in protein misfolding diseases and aging. Int. J. Cell Biol. 2013:638083 10.1155/2013/638083 24348562 PMC3855986

[B32] Cupers P. Orlans I. Craessaerts K. Annaert W. De Strooper B. (2001). The amyloid precursor protein (APP)-cytoplasmic fragment generated by γ-secretase is rapidly degraded but distributes partially in a nuclear fraction of neurones in culture. J. Neurochem. 78, 1168–1178 10.1046/j.1471-4159.2001.00516.x 11553691

[B33] Deane R. Wu Z. Zlokovic B. V. (2004). RAGE (yin) versus LRP (yang) balance regulates alzheimer amyloid beta-peptide clearance through transport across the blood-brain barrier. Stroke 35(11 Suppl. 1), 2628–2631 10.1161/01.str.0000143452.85382.d1 15459432

[B34] Deng Y. Lu X. Wang L. Li T. Ding Y. Cao H. (2014). Curcumin inhibits the AKT/NF-κB signaling via CpG Demethylation of the promoter and restoration of NEP in the N2a cell line. AAPS J. 16, 649–657 10.1208/s12248-014-9605-8 24756894 PMC4070258

[B35] Du J. Cho P. Y. Yang D. T. Murphy R. M. (2012). Identification of β-amyloid-binding sites on transthyretin. Protein Eng. Des. Sel. 25, 337–345 10.1093/protein/gzs026 22670059 PMC3530273

[B36] Du J. Zhang L. Liu S. Wang Z. (2010). Palmitic acid and docosahexaenoic acid opposingly regulate the expression of insulin-degrading enzyme in neurons. Pharmazie 65, 231–232 20383947

[B37] Eckman E. A. Adams S. K. Troendle F. J. Stodola B. A. Kahn M. A. Fauq A. H. (2006). Regulation of steady-state β-amyloid levels in the brain by neprilysin and endothelin-converting enzyme but not angiotensin-converting enzyme. J. Biol. Chem. 281, 30471–30478 10.1074/jbc.m605827200 16912050

[B38] Eckman E. A. Eckman C. B. (2005). Aβ-degrading enzymes: modulators of Alzheimer’s disease pathogenesis and targets for therapeutic intervention. Biochem. Soc. Trans. 33, 1101–1105 10.1042/bst20051101 16246055

[B39] Edbauer D. Willem M. Lammich S. Steiner H. Haass C. (2002). Insulin-degrading enzyme rapidly removes the beta-amyloid precursor protein intracellular domain (AICD). J. Biol. Chem. 277, 13389–13393 10.1074/jbc.m111571200 11809755

[B186] Eisele Y. S. Baumann M. Klebl B. Nordhammer C. Jucker M. Kilger E. (2007). Gleevec increases levels of the amyloid precursor protein intracellular domain and of the amyloid-β degrading enzyme neprilysin. Mol. Biol. Cell 18, 3591–3600 10.1091/mbc.e07-01-0035 17626163 PMC1951756

[B40] El-Amouri S. S. Zhu H. Yu J. Marr R. Verma I. M. Kindy M. S. (2008). Neprilysin: an enzyme candidate to slow the progression of Alzheimer’s disease. Am. J. Pathol. 172, 1342–1354 10.2353/ajpath.2008.070620 18403590 PMC2329843

[B41] Emoto N. Yanagisawa M. (1995). Endothelin-converting enzyme-2 is a membrane-bound, phosphoramidon-sensitive metalloprotease with acidic pH optimum. J. Biol. Chem. 270, 15262–15268 10.1074/jbc.270.25.15262 7797512

[B42] Famakin B. M. Mou Y. Johnson K. Spatz M. Hallenbeck J. (2014). A new role for downstream Toll-like receptor signaling in mediating immediate early gene expression during focal cerebral ischemia. J. Cereb. Blood Flow. Metab. 34, 258–267 10.1038/jcbfm.2013.182 24301291 PMC3915199

[B43] Fantini J. Di Scala C. Yahi N. Troadec J. D. Sadelli K. Chahinian H. (2014). Bexarotene blocks calcium-permeable ion channels formed by neurotoxic Alzheimer’s β-amyloid peptides. ACS Chem. Neurosci. 5, 216–224 10.1021/cn400183w 24383913 PMC4058752

[B44] Fernández-Gamba A. Leal M. C. Morelli L. Castaño E. M. (2009). Insulin-degrading enzyme: structure-function relationship and its possible roles in health and disease. Curr. Pharm. Des. 15, 3644–3655 10.2174/138161209789271799 19925417

[B45] Ferretti M. T. Partridge V. Leon W. C. Canneva F. Allard S. Arvanitis D. N. (2011). Transgenic mice as a model of pre-clinical Alzheimer’s disease. Curr. Alzheimer Res. 8, 4–23 10.2174/156720511794604561 21143159

[B46] Fisk L. Nalivaeva N. N. Boyle J. P. Peers C. S. Turner A. J. (2007). Effects of hypoxia and oxidative stress on expression of neprilysin in human neuroblastoma cells and rat cortical neurones and astrocytes. Neurochem. Res. 32, 1741–1748 10.1007/s11064-007-9349-2 17486446

[B47] Fisk L. Nalivaeva N. N. Turner A. J. (2006). Regulation of endothelin-converting enzyme-1 expression in human neuroblastoma cells. Exp. Biol. Med. (Maywood) 231, 1048–1053 16741047

[B48] Fujiwara H. Kimura J. Sakamoto M. Yokosuka A. Mimaki Y. Murata K. (2014). Nobiletin, a flavone from citrus depressa, induces gene expression and increases the protein level and activity of neprilysin in SK-N-SH cells. Can. J. Physiol. Pharmacol. 92, 351–355 10.1139/cjpp-2013-0440 24784468

[B50] Gahete M. D. Rubio A. Durán-Prado M. Avila J. Luque R. M. Castaño J. P. (2010). Expression of somatostatin, cortistatin and their receptors, as well as dopamine receptors, but not of neprilysin, are reduced in the temporal lobe of Alzheimer’s disease patients. J. Alzheimers Dis. 20, 465–475 10.3233/JAD-2010-1385 20164562

[B51] Garcia-Alloza M. Gregory J. Kuchibhotla K. V. Fine S. Wei Y. Ayata C. (2011). Cerebrovascular lesions induce transient β-amyloid deposition. Brain 134(Pt. 12), 3697–3707 10.1093/brain/awr300 22120142 PMC3235567

[B52] Grimm M. O. Mett J. Stahlmann C. P. Haupenthal V. J. Zimmer V. C. Hartmann T. (2013). Neprilysin and Aβ clearance: impact of the APP intracellular domain in NEP regulation and implications in Alzheimer’s disease. Front. Aging Neurosci. 5:98 10.3389/fnagi.2013.00098 24391587 PMC3870290

[B53] Guan H. Liu Y. Daily A. Police S. Kim M. H. Oddo S. (2009). Peripherally expressed neprilysin reduces brain amyloid burden: a novel approach for treating Alzheimer’s disease. J. Neurosci. Res. 87, 1462–1473 10.1002/jnr.21944 19021293 PMC2832596

[B54] Guerreiro R. J. Gustafson D. R. Hardy J. (2012). The genetic architecture of Alzheimer’s disease: beyond APP, PSENs and APOE. Neurobiol. Aging 33, 437–456 10.1016/j.neurobiolaging.2010.03.025 20594621 PMC2980860

[B55] Gupta A. Fujita W. Gomes I. Bobeck E. Devi L. A. (2014). Endothelin converting Enzyme-2 differentially regulates opioid receptor activity. Br. J. Pharmacol. [Epub ahead of print]. 10.1111/bph.12833 24990314 PMC4292980

[B56] Hafez D. Huang J. Y. Huynh A. M. Valtierra S. Rockenstein E. Bruno A. M. (2011). Neprilysin-2 is an important β-amyloid degrading enzyme. Am. J. Pathol. 178, 306–312 10.1016/j.ajpath.2010.11.012 21224067 PMC3069828

[B57] Hama E. Shirotani K. Masumoto H. Sekine-Aizawa Y. Aizawa H. Saido T. C. (2001). Clearance of extracellular and cell-associated amyloid β peptide through viral expression of neprilysin in primary neurons. J. Biochem. 130, 721–726 10.1093/oxfordjournals.jbchem.a003040 11726269

[B58] Haque R. Nazir A. (2014). Insulin-degrading enzyme: a link between Alzheimer’s and type 2 diabetes mellitus. CNS Neurol. Disord. Drug Targets 13, 259–264 10.2174/18715273113126660139 24059320

[B59] Hardy J. (2009). The amyloid hypothesis for Alzheimer’s disease: a critical reappraisal. J. Neurochem. 110, 1129–1134 10.1111/j.1471-4159.2009.06181.x 19457065

[B60] Hardy J. A. Higgins G. A. (1992). Alzheimer’s disease: the amyloid cascade hypothesis. Science 256, 184–185 10.1126/science.1566067 1566067

[B61] Hauser P. S. Ryan R. O. (2013). Impact of apolipoprotein E on Alzheimer’s disease. Curr. Alzheimer Res. 10, 809–817 10.2174/15672050113109990156 23919769 PMC3995977

[B62] Hayashi H. Tohda M. Watanabe H. Murakami Y. Matsumoto K. (2005). The effects of Choto-san on the mRNA expression of Alzheimer’s disease related factors in the permanent ischemic rat brain. Biol. Pharm. Bull. 28, 744–746 10.1248/bpb.28.744 15802822

[B63] Hemming M. L. Selkoe D. J. (2005). Amyloid β-protein is degraded by cellular angiotensin-converting enzyme (ACE) and elevated by an ACE inhibitor. J. Biol. Chem. 280, 37644–37650 10.1074/jbc.m508460200 16154999 PMC2409196

[B64] Henderson S. J. Andersson C. Narwal R. Janson J. Goldschmidt T. J. Appelkvist P. (2014). Sustained peripheral depletion of amyloid-β with a novel form of neprilysin does not affect central levels of amyloid-β. Brain 137, 553–564 10.1093/brain/awt308 24259408 PMC3914468

[B66] Hiltunen M. Mäkinen P. Peräniemi S. Sivenius J. van Groen T. Soininen H. (2009). Focal cerebral ischemia in rats alters APP processing and expression of Aβ peptide degrading enzymes in the thalamus. Neurobiol. Dis. 35, 103–113 10.1016/j.nbd.2009.04.009 19426802

[B67] Hoang M. V. Turner A. J. (1997). Novel activity of endothelin-converting enzyme: hydrolysis of bradykinin. Biochem. J. 327, 23–26 9355729 10.1042/bj3270023 PMC1218757

[B68] Howell S. Nalbantoglu J. Crine P. (1995). Neutral endopeptidase can hydrolyze beta-amyloid(1-40) but shows no effect on beta-amyloid precursor protein metabolism. Peptides 16, 647–652 10.1016/0196-9781(95)00021-b 7479298

[B69] Huang J. Y. Hafez D. M. James B. D. Bennett D. A. Marr R. A. (2012). Altered NEP2 expression and activity in mild cognitive impairment and Alzheimer’s disease. J. Alzheimers Dis. 28, 433–441 10.3233/JAD-2011-111307 22008264 PMC3320721

[B70] Iijima-Ando K. Hearn S. A. Granger L. Shenton C. Gatt A. Chiang H. C. (2008). Overexpression of neprilysin reduces alzheimer amyloid-β42 (Aβ42)-induced neuron loss and intraneuronal Aβ42 deposits but causes a reduction in cAMP-responsive element-binding protein-mediated transcription, age-dependent axon pathology and premature death in Drosophila. J. Biol. Chem. 283, 19066–19076 10.1074/JBC.M710509200 18463098 PMC2441542

[B71] Im H. Manolopoulou M. Malito E. Shen Y. Zhao J. Neant-Fery M. (2007). Structure of substrate-free human insulin-degrading enzyme (IDE) and biophysical analysis of ATP-induced conformational switch of IDE. J. Biol. Chem. 282, 25453–25463 10.1074/jbc.m701590200 17613531

[B72] Iwata N. Sekiguchi M. Hattori Y. Takahashi A. Asai M. Ji B. (2013). Global brain delivery of neprilysin gene by intravascular administration of AAV vector in mice. Sci. Rep. 3:1472 10.1038/srep01472 23503602 PMC3600598

[B73] Iwata N. Tsubuki S. Takaki Y. Shirotani K. Lu B. Gerard N. P. (2001). Metabolic regulation of brain Abeta by neprilysin. Science 292, 1550–1552 10.1126/science.1059946 11375493

[B74] Kalinin S. Richardson J. C. Feinstein D. L. (2009). A PPARδ agonist reduces amyloid burden and brain inflammation in a transgenic mouse model of Alzheimer’s disease. Curr. Alzheimer Res. 6, 431–437 10.2174/156720509789207949 19874267

[B75] Karch C. M. Jeng A. T. Nowotny P. Cady J. Cruchaga C. Goate A. M. (2012). Expression of novel Alzheimer’s disease risk genes in control and Alzheimer’s disease brains. PLoS One 7:e50976 10.1371/journal.pone.0050976 23226438 PMC3511432

[B76] Katsuda T. Tsuchiya R. Kosaka N. Yoshioka Y. Takagaki K. Oki K. (2013). Human adipose tissue-derived mesenchymal stem cells secrete functional neprilysin-bound exosomes. Sci. Rep. 3:1197 10.1038/srep01197 23378928 PMC3561625

[B77] Kerr M. A. Kenny A. J. (1974). The purification and specificity of a neutral endopeptidase from rabbit kidney brush border. Biochem. J. 137, 477–488 4423492 10.1042/bj1370477 PMC1166147

[B78] Kerridge C. Belyaev N. D. Nalivaeva N. N. Turner A. J. (2014). The Aβ-clearance protein transthyretin, like neprilysin, is epigenetically regulated by the amyloid precursor protein intracellular domain. J. Neurochem. 130, 419–431 10.1111/jnc.12680 24528201

[B79] Kiddle S. J. Sattlecker M. Proitsi P. Simmons A. Westman E. Bazenet C. (2014). Candidate blood proteome markers of Alzheimer’s disease onset and progression: a systematic review and replication study. J. Alzheimers Dis. 38, 515–531 10.3233/JAD-130380 24121966

[B80] Kilgore M. Miller C. A. Fass D. M. Hennig K. M. Haggarty S. J. Sweatt J. D. (2010). Inhibitors of class 1 histone deacetylases reverse contextual memory deficits in a mouse model of Alzheimer’s disease. Neuropsychopharmacology 35, 870–880 10.1038/npp.2009.197 20010553 PMC3055373

[B81] Kiss A. Kowalski J. Melzig M. F. (2006). Induction of neutral endopeptidase activity in PC-3 cells by an aqueous extract of Epilobium angustifolium L. and oenothein B. Phytomedicine 13, 284–289 10.1016/j.phymed.2004.08.002 16492533

[B82] Klein C. Patte-Mensah C. Taleb O. Bourguignon J. J. Schmitt M. Bihel F. (2013). The neuroprotector kynurenic acid increases neuronal cell survival through neprilysin induction. Neuropharmacology 70, 254–260 10.1016/j.neuropharm.2013.02.006 23422298

[B83] Kong Y. Ruan L. Qian L. Liu X. Le Y. (2010). Norepinephrine promotes microglia to uptake and degrade amyloid β peptide through upregulation of mouse formyl peptide receptor 2 and induction of insulin-degrading enzyme. J. Neurosci. 30, 11848–11857 10.1523/JNEUROSCI.2985-10.2010 20810904 PMC6633413

[B84] Konietzko U. (2012). AICD nuclear signaling and its possible contribution to Alzheimer’s disease. Curr. Alzheimer Res. 9, 200–216 10.2174/156720512799361673 21605035

[B85] Koudinov A. R. Berezov T. T. (2004). Alzheimer’s amyloid-β (Aβ) is an essential synaptic protein, not neurotoxic junk. Acta Neurobiol. Exp. (Wars) 64, 71–79 15190681 10.55782/ane-2004-1492

[B86] Kurochkin I. V. Goto S. (1994). Alzheimer’s beta-amyloid peptide specifically interacts with and is degraded by insulin degrading enzyme. FEBS Lett. 345, 33–37 10.1016/0014-5793(94)00387-4 8194595

[B87] Kurz A. Perneczky R. (2011). Amyloid clearance as a treatment target against Alzheimer’s disease. J. Alzheimers Dis. 24(Suppl. 2), 61–73 10.3233/JAD-2011-102139 21422524

[B88] LaFerla F. M. (2012). Preclinical success against Alzheimer’s disease with an old drug. N. Engl. J. Med. 367, 570–572 10.1056/NEJMcibr1204890 22873540

[B89] LaFerla F. M. Green K. N. (2012). Animal models of Alzheimer disease. Cold Spring Harb. Perspect. Med. 2:a006320 10.1101/cshperspect.a006320 23002015 PMC3543097

[B90] Lazarov O. Robinson J. Tang Y. P. Hairston I. S. Korade-Mirnics Z. Lee V. M. (2005). Environmental enrichment reduces Aβ levels and amyloid deposition in transgenic mice. Cell 120, 701–713 10.1016/j.cell.2005.01.015 15766532

[B91] Lee M. J. Lee J. H. Rubinsztein D. C. (2013). Tau degradation: the ubiquitin-proteasome system versus the autophagy-lysosome system. Prog. Neurobiol. 105, 49–59 10.1016/j.pneurobio.2013.03.001 23528736

[B92] Leissring M. A. Farris W. Chang A. Y. Walsh D. M. Wu X. Sun X. (2003). Enhanced proteolysis of β-amyloid in APP transgenic mice prevents plaque formation, secondary pathology and premature death. Neuron 40, 1087–1093 10.1016/s0896-6273(03)00787-6 14687544

[B93] Leissring M. A. Farris W. Wu X. Christodoulou D. C. Haigis M. C. Guarente L. (2004). Alternative translation initiation generates a novel isoform of insulin-degrading enzyme targeted to mitochondria. Biochem. J. 383, 439–446 10.1042/bj20041081 15285718 PMC1133736

[B94] Leissring M. A. Malito E. Hedouin S. Reinstatler L. Sahara T. Abdul-Hay S. O. (2010). Designed inhibitors of insulin-degrading enzyme regulate the catabolism and activity of insulin. PLoS One 5:e10504 10.1371/journal.pone.0010504 20498699 PMC2866327

[B95] Leissring M. A. Turner A. J. (2013). Regulation of distinct pools of amyloid β-protein by multiple cellular proteases. Alzheimers Res. Ther. 5:37 10.1186/alzrt194 23953275 PMC3978621

[B96] Letarte M. Vera S. Tran R. Addis J. B. Onizuka R. J. Quackenbush E. J. (1988). Common acute lymphocytic leukemia antigen is identical to neutral endopeptidase. J. Exp. Med. 168, 1247–1253 10.1084/jem.168.4.1247 2971756 PMC2189092

[B97] Li X. Ma Y. Wei X. Li Y. Wu H. Zhuang J. (2014). Clusterin in Alzheimer’s disease: a player in the biological behavior of amyloid-β. Neurosci. Bull. 30, 162–168 10.1007/s12264-013-1391-2 24353014 PMC5562576

[B98] Li X. Zhang X. Ladiwala A. R. Du D. Yadav J. K. Tessier P. M. (2013). Mechanisms of transthyretin inhibition of β-amyloid aggregation in vitro. J. Neurosci. 33, 19423–19433 10.1523/JNEUROSCI.2561-13.2013 24336709 PMC3858619

[B99] Lim C. S. Alkon D. L. (2014). PKCɛ promotes HuD-mediated neprilysin mRNA stability and enhances neprilysin-induced Aβ degradation in brain neurons. PLoS One 9:e97756 10.1371/journal.pone.0097756 24848988 PMC4029802

[B100] Lim H. J. Shim S. B. Jee S. W. Lee S. H. Lim C. J. Hong J. T. (2013). Green tea catechin leads to global improvement among Alzheimer’s disease-related phenotypes in NSE/hAPP-C105 Tg mice. J. Nutr. Biochem. 24, 1302–13013 10.1016/j.jnutbio.2012.10.005 23333093

[B102] Liu Y. Guan H. Beckett T. L. Juliano M. A. Juliano L. Song E. S. (2007). In vitro and in vivo degradation of Aβ peptide by peptidases coupled to erythrocytes. Peptides 28, 2348–2355 10.1016/j.peptides.2007.09.015 17988763 PMC2149904

[B101] Liu S. Liu J. Miura Y. Tanabe C. Maeda T. Terayama Y. (2014). Conversion of Aβ43 to Aβ40 by the successive action of angiotensin-converting enzyme 2 and angiotensin-converting enzyme. J. Neurosci. Res. 92, 1178–1186 10.1002/jnr.23404 24823497

[B103] Liu Y. Studzinski C. Beckett T. Guan H. Hersh M. A. Murphy M. P. (2009). Expression of neprilysin in skeletal muscle reduces amyloid burden in a transgenic mouse model of Alzheimer disease. Mol. Ther. 17, 1381–1386 10.1038/mt.2009.115 19471248 PMC2835241

[B104] Liu Y. Studzinski C. Beckett T. Murphy M. P. Klein R. L. Hersh L. B. (2010). Circulating neprilysin clears brain amyloid. Mol. Cell Neurosci. 45, 101–107 10.1016/j.mcn.2010.05.014 20558294 PMC2923273

[B105] Maetzler W. Stoycheva V. Schmid B. Schulte C. Hauser A. K. Brockmann K. (2010). Neprilysin activity in cerebrospinal fluid is associated with dementia and amyloid-β42 levels in Lewy body disease. J. Alzheimers Dis. 22, 933–938 10.3233/JAD-2010-101197 20858953

[B106] Maianti J. P. McFedries A. Foda Z. H. Kleiner R. E. Du X. Q. Leissring M. A. (2014). Anti-diabetic activity of insulin-degrading enzyme inhibitors mediated by multiple hormones. Nature 511, 94–98 10.1038/nature13297 24847884 PMC4142213

[B107] Mainardi M. Di Garbo A. Caleo M. Berardi N. Sale A. Maffei L. (2014). Environmental enrichment strengthens corticocortical interactions and reduces amyloid-β oligomers in aged mice. Front. Aging Neurosci. 6:1 10.3389/fnagi.2014.00001 24478697 PMC3899529

[B108] Maloney B. Lahiri D. K. (2011). The Alzheimer’s amyloid β-peptide (Aβ) binds a specific DNA Aβ-interacting domain (AβID) in the APP, BACE1 and APOE promoters in a sequence-specific manner: characterizing a new regulatory motif. Gene 488, 1–12 10.1016/j.gene.2011.06.004 21699964 PMC3381326

[B109] Marr R. A. Rockenstein E. Mukherjee A. Kindy M. S. Hersh L. B. Gage F. H. (2003). Neprilysin gene transfer reduces human amyloid pathology in transgenic mice. J. Neurosci. 23, 1992–1996 12657655 10.1523/JNEUROSCI.23-06-01992.2003 PMC6742010

[B110] Marr R. A. Spencer B. J. (2010). NEP-like endopeptidases and Alzheimer’s disease. Curr. Alzheimer Res. 7, 223–229 10.2174/156720510791050849 20088804

[B111] Martiskainen H. Haapasalo A. Kurkinen K. M. Pihlajamäki J. Soininen H. Hiltunen M. (2013). Targeting ApoE4/ApoE receptor LRP1 in Alzheimer’s disease. Expert Opin. Ther. Targets 17, 781–794 10.1517/14728222.2013.789862 23573918

[B112] Matsas R. Fulcher I. S. Kenny A. J. Turner A. J. (1983). Substance P and [Leu]enkephalin are hydrolyzed by an enzyme in pig caudate synaptic membranes that is identical with the endopeptidase of kidney microvilli. Proc. Natl. Acad. Sci. U S A 80, 3111–3115 10.1073/pnas.80.10.3111 6190172 PMC393984

[B113] Melino G. Draoui M. Bernardini S. Bellincampi L. Reichert U. Cohen P. (1996). Regulation by retinoic acid of insulin-degrading enzyme and of a related endoprotease in human neuroblastoma cell lines. Cell Growth Differ. 7, 787–796 8780892

[B114] Melzig M. F. Janka M. (2003). Enhancement of neutral endopeptidase activity in SK-N-SH cells by green tea extract. Phytomedicine 10, 494–498 10.1078/094471103322331449 13678233

[B115] Miners J. S. Baig S. Tayler H. Kehoe P. G. Love S. (2009). Neprilysin and insulin-degrading enzyme levels are increased in Alzheimer disease in relation to disease severity. J. Neuropathol. Exp. Neurol. 68, 902–914 10.1097/NEN.0b013e3181afe475 19606063

[B116] Miners J. S. Kehoe P. Love S. (2011). Neprilysin protects against cerebral amyloid angiopathy and Aβ-induced degeneration of cerebrovascular smooth muscle cells. Brain Pathol. 21, 594–605 10.1111/j.1750-3639.2011.00486.x 21382117 PMC8094282

[B117] Miners J. S. Van Helmond Z. Chalmers K. Wilcock G. Love S. Kehoe P. G. (2006). Decreased expression and activity of neprilysin in Alzheimer disease are associated with cerebral amyloid angiopathy. J. Neuropathol. Exp. Neurol. 65, 1012–1021 10.1097/01.jnen.0000240463.87886.9a 17021406

[B118] Morelli L. Llovera R. E. Alonso L. G. Frangione B. de Prat-Gay G. Ghiso J. (2005). Insulin-degrading enzyme degrades amyloid peptides associated with British and Danish familial dementia. Biochem. Biophys. Res. Commun. 332, 808–816 10.1016/j.bbrc.2005.05.020 15913558

[B119] Morita M. Kurochkin I. V. Motojima K. Goto S. Takano T. Okamura S. (2000). Insulin-degrading enzyme exists inside of rat liver peroxisomes and degrades oxidized proteins. Cell Struct. Funct. 25, 309–315 10.1247/csf.25.309 11235899

[B120] Mzhavia N. Pan H. Che F. Y. Fricker L. D. Devi L. A. (2003). Characterization of endothelin-converting enzyme-2. Implication for a role in the nonclassical processing of regulatory peptides. J. Biol. Chem. 278, 14704–14711 10.1074/jbc.m211242200 12560336 PMC3862352

[B121] Nalivaeva N. N. Babusikova E. Dobrota D. Turner A. J. (2005). Effect of ischaemia and reperfusion on the content and degradation of amyloid precursor protein in the hippocampus of rats. Neirokhimia 22, 207–212

[B122] Nalivaeva N. N. Beckett C. Belyaev N. D. Turner A. J. (2012a). Are amyloid-degrading enzymes viable therapeutic targets in Alzheimer’s disease? J. Neurochem. 120(Suppl. 1), 167–185 10.1111/j.1471-4159.2011.07510.x 22122230

[B123] Nalivaeva N. N. Belyaev N. D. Lewis D. I. Pickles A. R. Makova N. Z. Bagrova D. I. (2012b). Effect of sodium valproate administration on brain neprilysin expression and memory in rats. J. Mol. Neurosci. 46, 569–577 10.1007/s12031-011-9644-x 21932040

[B124] Nalivaeva N. N. Belyaev N. D. Turner A. J. (2009). Sodium valproate: an old drug with new roles. Trends Pharmacol. Sci. 30, 509–514 10.1016/j.tips.2009.07.002 19762089

[B125] Nalivaeva N. N. Belyaev N. D. Zhuravin I. A. Turner A. J. (2012c). The Alzheimer’s amyloid-degrading peptidase, neprilysin: can we control it? Int. J. Alzheimers Dis. 2012:383796 10.1155/2012/383796 22900228 PMC3412116

[B126] Nalivaeva N. N. Fisk L. Kochkina E. G. Plesneva S. A. Zhuravin I. A. Babusikova E. (2004). Effect of hypoxia/ischemia and hypoxic preconditioning/reperfusion on expression of some amyloid-degrading enzymes. Ann. N Y Acad. Sci. 1035, 21–33 10.1196/annals.1332.002 15681798

[B127] Nalivaeva N. N. Turner A. J. (2013). The amyloid precursor protein: a biochemical enigma in brain development, function and disease. FEBS Lett. 587, 2046–2054 10.1016/j.febslet.2013.05.010 23684647

[B128] Noh H. Seo H. (2014). Age-dependent effects of valproic acid in Alzheimer’s disease (AD) mice are associated with nerve growth factor (NGF) regulation. Neuroscience 266, 255–265 10.1016/j.neuroscience.2014.02.012 24568732

[B129] Nunes A. F. Saraiva M. J. Sousa M. M. (2006). Transthyretin knockouts are a new mouse model for increased neuropeptide Y. FASEB J. 20, 166–168 16263939 10.1096/fj.05-4106fje

[B130] Pacheco-Quinto J. Eckman E. A. (2013). Endothelin-converting enzymes degrade intracellular β-amyloid produced within the endosomal/lysosomal pathway and autophagosomes. J. Biol. Chem. 288, 5606–5615 10.1074/jbc.M112.422964 23283972 PMC3581387

[B131] Pacheco-Quinto J. Herdt A. Eckman C. B. Eckman E. A. (2013). Endothelin-converting enzymes and related metalloproteases in Alzheimer’s disease. J. Alzheimers Dis. 33(Suppl. 1), S101–S110 22903130 10.3233/JAD-2012-129043 PMC3677825

[B132] Palmer J. C. Baig S. Kehoe P. G. Love S. (2009). Endothelin-Converting Enzyme-2 is increased in Alzheimer’s disease and up-regulated by Aβ. Am. J. Pathol. 175, 262–270 10.2353/ajpath.2009.081054 19541930 PMC2708812

[B133] Palmer J. C. Barker R. Kehoe P. G. Love S. (2012). Endothelin-1 is elevated in Alzheimer’s disease and upregulated by amyloid-β. J. Alzheimers Dis. 29, 853–861 10.3233/JAD-2012-111760 22330820

[B134] Palmer J. C. Tayler H. M. Love S. (2013). Endothelin-converting enzyme-1 activity, endothelin-1 production and free radical-dependent vasoconstriction in Alzheimer’s disease. J. Alzheimers Dis. 36, 577–587 10.3233/JAD-130383 23629587

[B135] Papadopoulos P. Tong X. K. Hamel E. (2014). Selective benefits of simvastatin in bitransgenic APPSwe,Ind/TGF-β1 mice. Neurobiol. Aging 35, 203–212 10.1016/j.neurobiolaging.2013.07.010 23954171

[B136] Pardossi-Piquard R. Checler F. (2012). The physiology of the β-amyloid precursor protein intracellular domain AICD. J. Neurochem. 120(Suppl. 1), 109–124 10.1111/j.1471-4159.2011.07475.x 22122663

[B137] Pardossi-Piquard R. Petit A. Kawarai T. Sunyach C. Alves da Costa C. Vincent B. (2005). Presenilin-dependent transcriptional control of the Aβ-degrading enzyme neprilysin by intracellular domains of βAPP and APLP. Neuron 46, 541–554 10.1016/j.neuron.2005.04.008 15944124

[B138] Passer B. Pellegrini L. Russo C. Siegel R. M. Lenardio M. J. Schettini G. (2000). Generation of an apoptotic intracellular peptide by γ-secretase cleavage of Alzheimer’s amyloid β protein precursor. J. Alzheimers Dis. 2, 289–301 12214090 10.3233/jad-2000-23-408

[B139] Pluta R. Jabłoński M. Ułamek-Kozioł M. Kocki J. Brzozowska J. Januszewski S. (2013). Sporadic Alzheimer’s disease begins as episodes of brain ischemia and ischemically dysregulated Alzheimer’s disease genes. Mol. Neurobiol. 48, 500–515 10.1007/s12035-013-8439-1 23519520 PMC3825141

[B140] Pope D. Madura J. D. Cascio M. (2014). β-Amyloid and neprilysin computational studies identify critical residues implicated in binding specificity. J. Chem. Inf. Model. 54, 1157–1165 10.1021/ci500015m 24650257

[B141] Qing H. He G. Ly P. T. Fox C. J. Staufenbiel M. Cai F. (2008). Valproic acid inhibits Aβ production, neuritic plaque formation and behavioral deficits in Alzheimer’s disease mouse models. J. Exp. Med. 205, 2781–2789 10.1084/jem.20081588 18955571 PMC2585842

[B142] Qiu W. Q. Folstein M. F. (2006). Insulin, insulin-degrading enzyme and amyloid-β peptide in Alzheimer’s disease: review and hypothesis. Neurobiol. Aging 27, 190–198 10.1016/j.neurobiolaging.2005.01.004 16399206

[B143] Roques B. P. Fournié-Zaluski M. C. Soroca E. Lecomte J. M. Malfroy B. Llorens C. (1980). The enkephalinase inhibitor thiorphan shows antinociceptive activity in mice. Nature 288, 286–288 10.1038/288286a0 7001254

[B144] Saido T. C. (2013). Metabolism of amyloid β peptide and pathogenesis of Alzheimer’s disease. Proc. Jpn. Acad. Ser. B Phys. Biol. Sci. 89, 321–339 10.2183/pjab.89.321 23883611 PMC3758963

[B145] Saito T. Iwata N. Tsubuki S. Takaki Y. Takano J. Huang S. M. (2005). Somatostatin regulates brain amyloid β peptide Aβ42 through modulation of proteolytic degradation. Nat. Med. 11, 434–439 10.1038/nm1206 15778722

[B148] Sattlecker M. Kiddle S. J. Newhouse S. Proitsi P. Nelson S. Williams S. (2014). Alzheimer’s disease biomarker discovery using SOMAscan multiplexed protein technology. Alzheimers Dement. [Epub ahead of print]. 10.1016/j.jalz.2013.09.016 24768341

[B149] Schroeter E. H. Kisslinger J. A. Kopan R. (1998). Notch-1 signalling requires ligand-induced proteolytic release of intracellular domain. Nature 393, 382–386 10.1038/30756 9620803

[B152] Shirotani K. Tsubuki S. Iwata N. Takaki Y. Harigaya W. Maruyama K. (2001). Neprilysin degrades both amyloid β peptides 1-40 and 1-42 most rapidly and efficiently among thiorphan- and phosphoramidon-sensitive endopeptidases. J. Biol. Chem. 276, 21895–21901 10.1074/jbc.m008511200 11278416

[B153] Smith A. M. Gibbons H. M. Dragunow M. (2010). Valproic acid enhances microglial phagocytosis of amyloid-β(1-42). Neuroscience 169, 505–515 10.1016/j.neuroscience.2010.04.041 20423723

[B154] Sousa J. C. Cardoso I. Marques F. Saraiva M. J. Palha J. A. (2007). Transthyretin and Alzheimer’s disease: where in the brain? Neurobiol. Aging 28, 713–718 10.1016/j.neurobiolaging.2006.03.015 16698124

[B155] Spencer B. Marr R. A. Gindi R. Potkar R. Michael S. Adame A. (2011). Peripheral delivery of a CNS targeted, metalo-protease reduces Aβ toxicity in a mouse model of Alzheimer’s disease. PLoS One 6:e16575 10.1371/journal.pone.0016575 21304989 PMC3031588

[B156] Spencer B. Verma I. Desplats P. Morvinski D. Rockenstein E. Adame A. (2014). A neuroprotective brain-penetrating endopeptidase fusion protein ameliorates Alzheimer disease pathology and restores neurogenesis. J. Biol. Chem. 289, 17917–17931 10.1074/jbc.m114.557439 24825898 PMC4067222

[B157] Stein T. D. Johnson J. A. (2002). Lack of neurodegeneration in transgenic mice overexpressing mutant amyloid precursor protein is associated with increased levels of transthyretin and the activation of cell survival pathways. J. Neurosci. 22, 7380–7388 12196559 10.1523/JNEUROSCI.22-17-07380.2002 PMC6758007

[B158] Strittmatter W. J. (2012). Medicine. Old drug, new hope for Alzheimer’s disease. Science 335, 1447–1448 10.1126/science.1220725 22442467

[B159] Tamboli I. Y. Barth E. Christian L. Siepmann M. Kumar S. Singh S. (2010). Statins promote the degradation of extracellular amyloid β-peptide by microglia via stimulation of exosome-associated insulin-degrading enzyme (IDE) secretion. J. Biol. Chem. 285, 37405–37414 10.1074/jbc.M110.149468 20876579 PMC2988346

[B160] Tariot P. N. Aisen P. S. (2009). Can lithium or valproate untie tangles in Alzheimer’s disease? J. Clin. Psychiatry 70, 919–921 10.4088/jcp.09com05331 19573485

[B161] Tseng B. P. Green K. N. Chan J. L. Blurton-Jones M. LaFerla F. M. (2008). Aβ inhibits the proteasome and enhances amyloid and tau accumulation. Neurobiol. Aging 29, 1607–1618 10.1016/j.neurobiolaging.2007.04.014 17544172 PMC2664168

[B162] Tsuzuki K. Fukatsu R. Yamaguchi H. Tateno M. Imai K. Fujii N. (2000). Transthyretin binds amyloid β peptides, Aβ1-42 and Aβ1-40 to form complex in the autopsied human kidney - possible role of transthyretin for Aβ sequestration. Neurosci. Lett. 281, 171–174 10.1016/s0304-3940(00)00834-x 10704770

[B163] Turner A. J. Belyaev N. D. Nalivaeva N. N. (2011). Mediator: the missing link in amyloid precursor protein nuclear signalling. EMBO Rep. 12, 180–181 10.1038/embor.2011.25 21368841 PMC3059916

[B187] Vázquez M. C. Vargas L. M. Inestrosa N. C. Alvarez A. R. (2009). c-Abl modulates AICD dependent cellular responses: transcriptional induction and apoptosis. J. Cell. Physiol. 220, 136–143 10.1002/jcp.21743 19306298

[B164] Vieira M. Saraiva M. J. (2013). Transthyretin regulates hippocampal 14-3-3ζ protein levels. FEBS Lett. 587, 1482–1488 10.1016/j.febslet.2013.03.011 23523922

[B165] Vieira M. Saraiva M. J. (2014). Transthyretin: a multifaceted protein. Biomol. Concepts 5, 45–54 10.1515/bmc-2013-0038 25372741

[B166] Vingtdeux V. Hamdane M. Begard S. Loyens A. Delacourte A. Beauvillain J. C. (2007). Intracellular pH regulates amyloid precursor protein intracellular domain accumulation. Neurobiol. Dis. 25, 686–696 10.1016/j.nbd.2006.09.019 17207630

[B167] Walker J. R. Pacoma R. Watson J. Ou W. Alves J. Mason D. E. (2013). Enhanced proteolytic clearance of plasma Aβ by peripherally administered neprilysin does not result in reduced levels of brain Aβ in mice. J. Neurosci. 33, 2457–2464 10.1523/jneurosci.3407-12.2013 23392674 PMC6619149

[B168] Walsh D. M. Klyubin I. Fadeeva J. V. Cullen W. K. Anwyl R. Wolfe M. S. (2002). Naturally secreted oligomers of amyloid beta protein potently inhibit hippocampal long-term potentiation in vivo. Nature 416, 535–539 10.1038/416535a 11932745

[B169] Wang X. Cattaneo F. Ryno L. Hulleman J. Reixach N. Buxbaum J. N. (2014a). The systemic amyloid precursor transthyretin (TTR) behaves as a neuronal stress protein regulated by HSF1 in SH-SY5Y human neuroblastoma cells and APP23 Alzheimer’s disease model mice. J. Neurosci. 34, 7253–7265 10.1523/jneurosci.4936-13.2014 24849358 PMC4028500

[B170] Wang C. Sun B. Zhou Y. Grubb A. Gan L. (2012). Cathepsin B degrades amyloid-β in mice expressing wild-type human amyloid precursor protein. J. Biol. Chem. 287, 39834–39841 10.1074/jbc.m112.371641 23024364 PMC3501032

[B171] Wang S. Wang R. Chen L. Bennett D. A. Dickson D. W. Wang D. S. (2010). Expression and functional profiling of neprilysin, insulin-degrading enzyme and endothelin-converting enzyme in prospectively studied elderly and Alzheimer’s brain. J. Neurochem. 115, 47–57 10.1016/j.jalz.2010.08.173 20663017 PMC2939954

[B173] Wang Z. Zhang X. J. Li T. Li J. Tang Y. Le W. (2014b). Valproic acid reduces neuritic plaque formation and improves learning deficits in APP(Swe) /PS1(A246E) transgenic mice via preventing the prenatal hypoxia-induced down-regulation of neprilysin. CNS Neurosci. Ther. 20, 209–217 10.1111/cns.12186 24289518 PMC6493036

[B175] Watanabe H. Zhao Q. Matsumoto K. Tohda M. Murakami Y. Zhang S. H. (2003). Pharmacological evidence for antidementia effect of Choto-san (Gouteng-san), a traditional Kampo medicine. Pharmacol. Biochem. Behav. 75, 635–643 10.1016/s0091-3057(03)00109-6 12895681

[B176] Webster C. I. Burrell M. Olsson L. L. Fowler S. B. Digby S. Sandercock A. (2014). Engineering neprilysin activity and specificity to create a novel therapeutic for Alzheimer’s disease. PLoS One 9:e104001 10.1371/journal.pone.0104001 25089527 PMC4121237

[B177] Whyteside A. R. Turner A. J. (2008). Human neprilysin-2 (NEP2) and NEP display distinct subcellular localisations and substrate preferences. FEBS Lett. 582, 2382–2386 10.1016/j.febslet.2008.05.046 18539150

[B178] Xu X. Zhou H. Boyer T. G. (2011). Mediator is a transducer of amyloid-precursor-protein-dependent nuclear signalling. EMBO Rep. 12, 216–222 10.1038/embor.2010.210 21293490 PMC3059912

[B179] Yang Y. Song W. (2013). Molecular links between Alzheimer’s disease and diabetes mellitus. Neuroscience 250, 140–150 10.1016/j.neuroscience.2013.07.009 23867771

[B180] Yang H. Xie Z. Wei L. Yang H. Yang S. Zhu Z. (2013). Human umbilical cord mesenchymal stem cell-derived neuron-like cells rescue memory deficits and reduce amyloid-beta deposition in an AβPP/PS1 transgenic mouse model. Stem Cell Res. Ther. 4:76 10.1186/scrt227 23826983 PMC3854736

[B181] Yasojima K. Akiyama H. McGeer E. G. McGeer P. L. (2001). Reduced neprilysin in high plaque areas of Alzheimer’s brain: a possible relationship to deficient degradation of β-amyloid peptide. Neurosci. Lett. 297, 97–100 10.1016/s0304-3940(00)01675-x 11121879

[B182] Yu J. T. Tan L. (2012). The role of clusterin in Alzheimer’s disease: pathways, pathogenesis and therapy. Mol. Neurobiol. 45, 314–326 10.1007/s12035-012-8237-1 22274961

[B183] Zhao J. Li L. Leissring M. A. (2009). Insulin-degrading enzyme is exported via an unconventional protein secretion pathway. Mol. Neurodegener. 4:4 10.1186/1750-1326-4-4 19144176 PMC2632642

[B184] Zou K. Yamaguchi H. Akatsu H. Sakamoto T. Ko M. Mizoguchi K. (2007). Angiotensin-converting enzyme converts amyloid β beta-protein 1-42 (Aβ(1-42)) to Aβ(1-40) and its inhibition enhances brain Aβ deposition. J. Neurosci. 27, 8628–8635 10.1523/jneurosci.1549-07.2007 17687040 PMC6672927

[B185] Zraika S. Aston-Mourney K. Marek P. Hull R. L. Green P. S. Udayasankar J. (2010). Neprilysin impedes islet amyloid formation by inhibition of fibril formation rather than peptide degradation. J. Biol. Chem. 285, 18177–18183 10.1074/jbc.m109.082032 20400513 PMC2881741

